# Wild Edible Plants Used by the Polish Community in Misiones, Argentina

**DOI:** 10.1007/s10745-015-9790-9

**Published:** 2015-11-24

**Authors:** Monika Kujawska, Łukasz Łuczaj

**Affiliations:** Institute of Ethnology and Cultural Anthropology, University of Lodz, Pomorska 149/153, 90-236 Lodz, Poland; Department of Botany, Institute of Applied Biotechnology and Basic Science, University of Reszów, Werynia 502, 36-100 Kolbuszowa, Poland

**Keywords:** Cross-cultural ethnobotany, Wild food plants, Cultural significance indices, Polish migrants, Atlantic Forest, Argentina

## Abstract

We studied the cultural significance of wild edible plants for Eastern European migrants who settled in rural subtropical areas of South America. In 50 interviews with Polish migrants and their descendants in northern Misiones, Argentina, we recorded the use of 41 botanical species and two mushroom taxa. Different cultural significance indices were applied and sociodemographic factors such as gender, age and origin were addressed. Out of the ten most salient species, nine were fruits (*Eugenia uniflora*, *Eugenia involucrata*, *Rollinia salicifolia*, *Campomanesia xanthocarpa*, *Syagrus romanzoffiana*, *Allophylus edulis*, *Plinia peruviana*, *Plinia rivularis*, *Eugenia pyriformis*) and only one was a green vegetable (*Hypochaeris chillensis*). None of our informants reported famine foods, recreational teas or condiments. Men mentioned more wild edible species than women due to their more extensive knowledge of the forest plants growing further from settlements.

## Introduction

Diverse factors such as modes of subsistence, economy, ecology and cultural attitudes shape people’s knowledge and use of wild edible plants. It is well acknowledged that in urban centers people tend to use fewer wild edible plants than in rural areas due to limited access to such resources. The contemporary fashion for wild edible plants among city dwellers is usually limited to easily identifiable and readily available species (Nolan 2001; Kujawska and Łuczaj 2010; Łuczaj *et al.*^2012^), with some specialized exceptions (Poe *et al*. 2013).

Since agriculturists use both cultivated and wild species, especially in times of food shortage and social unrest (Brown 1985; Huss-Ashmore and Johnston 1994), the distinction between cultivators and foragers is not obvious. Nevertheless, both access to wild resources and local plant diversity determine the intensity of their use (Ladio and Lozada 2004).

The process of enculturation^1^ is to a large extent responsible for the establishment of food preferences (Johns 1994). For example, attitudes towards wild green vegetables differ throughout the world. We define green vegetables as the green parts of plants, such as leaves, stalks, buds and immature fruits, which are eaten either raw (as salads or snacks) or cooked. We also define exotic species or non-tended garden escapees to be “wild” (for an extended definition of wild foods see, e.g., Łuczaj *et al*. 2012). The predominance of wild green vegetables among gathered food types has been observed in many societies in Eastern Asia, some parts of Africa, Argentinean Patagonia, Mexico and the Mediterranean region (Bye 1981; Pieroni 2001; Ladio and Lozada 2003, 2004; Delang 2006; Leonti *et al*. 2006; Cruz-García and Price 2012; Kang *et al*. 2013, 2014; Łuczaj *et al*. 2013). In one small region of China, Kang and colleagues (2013) recorded the use of 126 green vegetables among the local communities of the Houzhenzi and Dali valleys*.* In contrast horticulturist and forager groups from the tropics and subtropics of South America display virtually no interest in wild greens, instead using sporadically leafy vegetables from their home gardens and plots as emergency food (Dufour and Wilson 1994; Aguilar and Condit 2001; Arenas and Scarpa 2007; Katz *et al.*^2012^). Poles are somewhere in between these two extremes. Most of the wild greens used in the Polish countryside have been famine plants and children’s snacks. Hence, the dramatic decrease in the use of wild greens in Poland observed after World War II is associated with rejection of these plants as poverty food and with changes in modes of subsistence in the countryside (Łuczaj 2008, 2010; Kujawska and Łuczaj 2010). A similar disregard for wild greens is observed in some parts of Latin America among the Mestizo population (Pelto *et al.*^1989^; Arias Toledo *et al.*^2007^). Łuczaj (2010) and Katz *et al.* (2012) noted that the lack of interest in gathering wild greens may be coupled with the low cultural status of all green vegetables (including cultivated ones).

Within a given culture, gender and age may also be important factors shaping knowledge and use of wild food resources. There are many studies that indicate differences between men and women in their explorations of certain environments in obtaining wild edible plants (Howard 2003; Pieroni 2003; Turner 2003; Kang *et al.*^2013^).

To date researchers have devoted little attention to processes of change in the use of wild edible plants by migrant groups.^2^ Most research has focused on medicinal plant use among migrant communities (Pieroni and Vandebroek 2007; Pirker *et al.*^2012^; Haselmair *et al.*^2014^). Our study of changes in the use of wild edible plants is unique as it was carried out among migrants originating from a European temperate climate who settled in the subtropics of a different continent, South America.

In the migratory process some traditional practices may gain greater significance for migrants than they had in their country of origin. This phenomenon is explained by the fact that migrants frequently adhere to some native customs and traditions in their search for a new identity in the host country (Nguyen 2003; Pieroni and Vandebroek 2007). Assessing the cultural salience of wild edible plants in a given society/community may also be important for a better understanding of the significance of these resources for the community.

The aims of this study were therefore 1) to record wild edible plants used by Polish migrants in Misiones, Argentina, 2) to analyze the cultural salience of these resources, and 3) to see how the use of wild plants is influenced by sociodemographic factors such as age, gender and origin, as well as plant accessibility.

## Methods

### Study Site

Misiones is one of the smallest, greenest and most biologically diverse Argentinean provinces. As a part of a greater ecoregion known as the Atlantic Forest of the Upper Parana (*la Selva paranaense*) (Plací and Di Bitetti 2006), it is home to 3000 vascular plant species At the end of the nineteenth century this ecoregion extended over 1.2 million km^2^ from the Paraguay River to the Atlantic Ocean, covering eastern Paraguay, southern Brazil and the province of Misiones in Argentina. During the twentieth century, the expansion of agriculture and animal husbandry, as well as deforestation, reduced it to 7.8 % of its former size (Ferrero 2005). Misiones has preserved the largest continuous corridor of the Atlantic Forest in the Upper Parana: according to different sources, 25 % (Moreau 2006) or 20 % of its total size (Ferrero 2005). The forest region covers 80 % of Misiones, extending through its central and northern parts where the dominant vegetation is subtropical, semi-deciduous. Summer, between December and March, has temperatures around 35–40 °C, and winter, between June and August, has frosts and temperatures between 0 and 18 *°*C. The mean annual temperature is 20.7 *°*C. Average annual rainfall is 1700–2200 mm, with no marked dry season (Crespo 1982).

The environmental wealth of Misiones is threatened by human activity and expansion. Prior to 1767, the region was part of the ‘theocratic empire’ of the Jesuit missions, which gave their name to the modern province. The missions were self-sustaining political, religious and economic organizations engaged in the evangelization and acculturation of the indigenous population of the Tupi-Guarani linguistic family. With the expulsion of the Jesuits in 1767, the region of present-day Misiones was nearly abandoned by the indigenous Guarani people. Throughout the nineteenth century the area was used for logging, yerba mate (*Ilex paraguariensis* A.St. –Hil.) extraction, and livestock pasturing in the south. In 1897, the first European immigrants arrived in the south of Misiones. They were of Polish and Ukrainian origin, having come from Galicja (Galizien), which was then the northern province of the Austro-Hungarian empire (Bartolomé 1982; Stemplowski 1991). The process of populating the province with European peasant families continued until the 1940s, varying in character but basically relying on an ethnic pattern of settlement. Land parcels of 100 hectares and later of 25 hectares were granted to immigrant families (Ferrero 2005). The large variety of ethnic groups coexisting in one of the smallest Argentinean provinces made it an extremely multicultural region.

At present the most important economic activities in the region are forestry, agriculture and, to a lesser extent, cattle breeding. Forestry is based on monoculture plantations of exotic species of pine (*Pinus* spp.) and eucalyptus (*Eucalyptus* spp.) for the paper and timber industries. The main crops are tobacco (*Nicotiana tabacum* L.), yerba mate, tea (*Thea sinensis* L.), and citrus (*Citrus* spp.). The local economy is based on exploitation of raw materials with little industrial development (Schiavoni 1998).

**Fig. 1:**
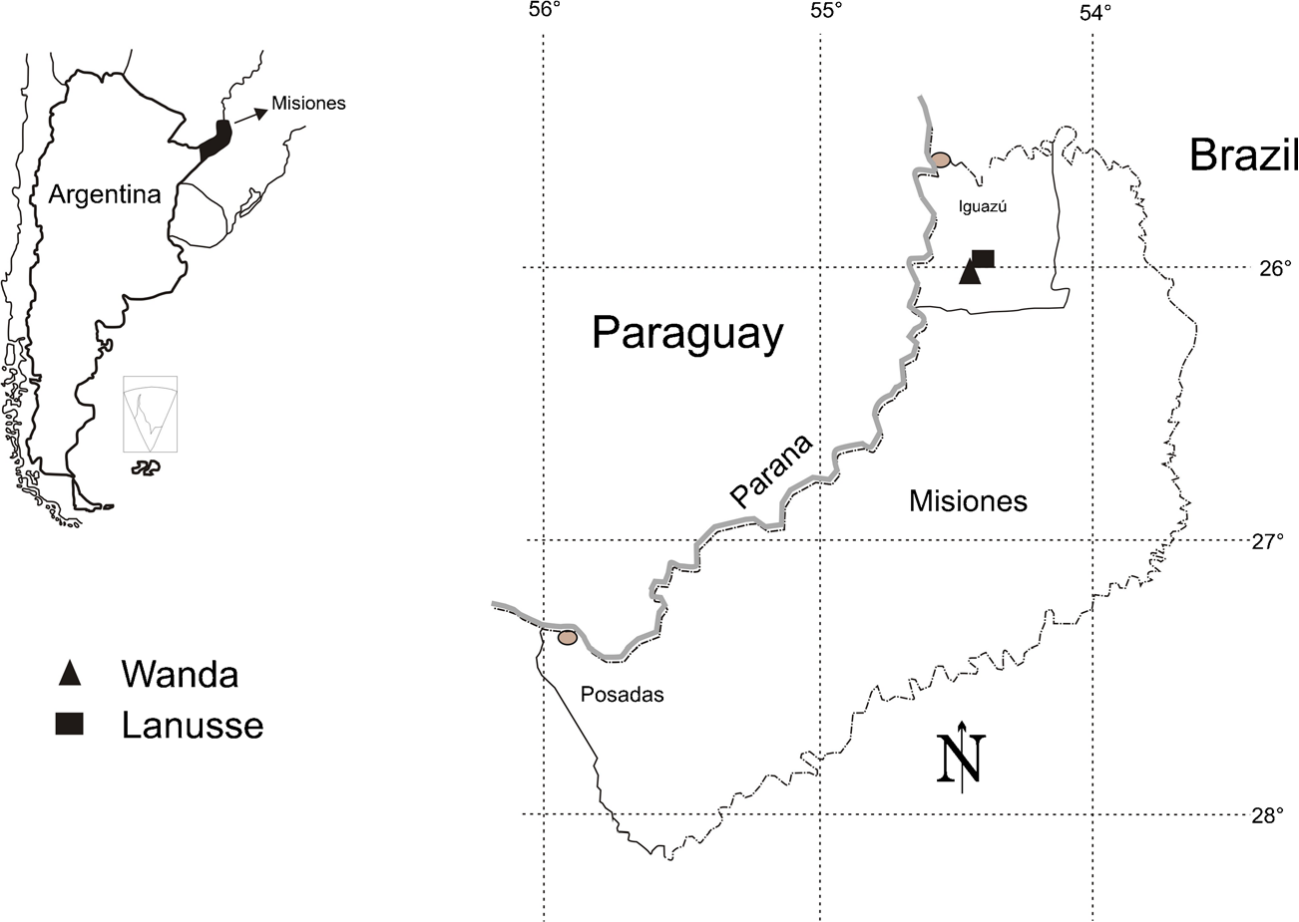
Polish settlements located in the north of the province of Misiones, Argentina

This study of wild food plants was undertaken in two localities in northern Misiones (Fig. 1): Wanda and Gobernador J.J. Lanusse (hereinafter Lanusse) between March and June of 2011. The settlements were founded as Polish agricultural colonies, in 1936 and 1937 respectively, by Colonizadora del Norte, a private company. The Wanda colony (25°58′S, 54°27′W) situated on the banks of the Parana and close to National Highway 12, developed into a town with some semi-rural parcels on its outskirts. The Lanusse colony (26°S, 54°28′W) 36 km from Wanda, never managed to develop administrative, educational or public health infrastructure. As a result, many families left Lanusse and moved to Wanda while continuing to use their land in Lanusse. At present, Wanda and Lanusse are multicultural places, inhabited by Paraguayan and Brazilian immigrants in addition to Argentineans and Polish settlers and their descendants.

### Study Group

The study group consisted of Polish migrants and their offspring, first and second generation born in Misiones. Altogether, 50 people were interviewed: 27 women and 23 men. The mean age of women participants was 61 ± 12 SD (30 to 83), and male participants, 62 ± 9 (43 to 75). The study population was divided into three groups: people who came directly from Poland; people whose parents and grandparents came from Poland; and those who first migrated to Brazil and only later to Argentina, eventually settling in Wanda and Lanusse.

In total, Lanusse has 150 inhabitants. Although there are no official statistics, we counted 16 families of Polish origin—approximately 60 people including children. We interviewed 15 persons from this group, of whom at least 50 % were heads of household (male or female). In a few cases we interviewed both husband and wife if we knew that they had different origins, e.g., one of them had parents who came to Misiones from Brazil and the other came straight from Poland.

Wanda has a total population of 16,000. There are no official statistics as to the number of people of Polish origin. We used informal estimates and a list of families provided by the Polish Association of Wanda. According to this list, there are 250 families of Polish origin—approximately 1200 people, including mixed couples (i.e., one non—Polish spouse). For the Wanda study we interviewed 35 persons, of whom at least 15 % were heads of household (male or female). Most interviewees in Wanda had once lived in Lanusse and moved to Wanda within the last 20 years. In some cases they were related to each other, as intermarrying prevailed among Polish settlers, especially until the mid 1970s. For sampling purposes we used a list of Polish families with their addresses in Wanda and Lanusse. We interviewed all those who were willing to spare their time and share their knowledge. It was not our intention to interview the most knowledgeable people in the community. Our aim was only to have a similar number of women and men for reasons of comparison (for more information about the Polish community in Misiones see also Kujawska 2010; Kujawska *et al.*^2012^; Kujawska and Hilgert 2014). Prior informed consent was obtained verbally before commencing each interview and the required permissions for data and voucher specimen collection were obtained from the Provincial Ministry of Ecology in Posadas, Misiones. The research was carried out following the code of ethics of the American Anthropological Association and the International Society of Ethnobiology Code of Ethics.

### Field Methods and Voucher Collection

We employed the free listing technique for obtaining information about wild edible plants (Quinlan 2005). The study participants were first asked to elicit all the wild edible plants they had ever eaten in their life. Then for all the mentioned taxa the following questions were asked:

1. Is this species very common, common, of medium frequency, or rare?
2. When was the last time that you tried this plant?
3. Which plant parts have you consumed?
4. How do you use and prepare this plant?
5. How much do you appreciate this plant? Please give your score between 1 and 10.
6. Does this plant have any medicinal properties, and if so, which part is used as medicine?

These free listing structured interviews were complemented with biographical interviews as well as with participant observation.

The free listed plants were collected in the presence of the study participants in Wanda and Lanusse. Then voucher specimens were dried and identified by the first author (MK) and deposited in the herbarium CTES of the Instituto de Botánica del Nordeste in Corrientes and Museo Nacional de Ciencias Naturales “Bernardino Rivadavia” in Buenos Aires (BA).

### Data Analysis

Only taxa whose use was confirmed by at least two study participants were included in the analysis. In order to measure the cultural importance of particular wild foods we used three indices. The Citation Index, the Salience Index and the Cultural Food Significance Index (CFSI) developed by Pieroni (2001) in his study of wild foods in Italy.

Salience was estimated using Smith’s Salience Index (Smith 1993). The index for species A is the mean of the following ratio calculated for each free listed plant:

$$ \mathrm{Salience}\;\mathrm{Index}=\frac{\mathrm{total}\;\mathrm{no}.\;\mathrm{o}\mathrm{f}\;\mathrm{item}\mathrm{s}\;\mathrm{in}\;\mathrm{a}\;\mathrm{list}\hbox{-} \mathrm{rank}\;\mathrm{o}\mathrm{r}\mathrm{der}\;\mathrm{o}\mathrm{f}\;\mathrm{s}\mathrm{pecies}\;\mathrm{A}\;\left(\mathrm{starting}\;\mathrm{f}\mathrm{r}\mathrm{o}\mathrm{m}\;0\;\mathrm{f}\mathrm{o}\mathrm{r}\;\mathrm{t}\mathrm{he}\;1\mathrm{s}\mathrm{t}\;\mathrm{item}\right)}{\mathrm{total}\;\mathrm{number}\;\mathrm{o}\mathrm{f}\;\mathrm{item}\mathrm{s}\;\mathrm{in}\;\mathrm{a}\;\mathrm{list}} $$

Thus a species consistently cited first gets an index of 1 and the items cited at the end of the free list tend to have Smith’s indices close to 0.

The cultural food significance index (CFSI) was elaborated specifically to evaluate the cultural significance of wild edibles. The CFSI was calculated according to Pieroni (2001) as

$$ \mathrm{CFSI}=\mathrm{QI}\ \mathrm{x}\ \mathrm{AI}\ \mathrm{x}\ \mathrm{F}\mathrm{U}\mathrm{I}\ \mathrm{x}\ \mathrm{P}\mathrm{U}\mathrm{I}\ \mathrm{x}\ \mathrm{MFFI}\ \mathrm{x}\ \mathrm{TSAI}\ \mathrm{x}\ \mathrm{F}\mathrm{MRI}\ \mathrm{x}\ {10}^{-2} $$

The formula takes into account seven indices: frequency of quotation (QI, ranging from 2 to the total number of informants), availability (AI, ranging from 0 to 4), frequency of utilization (FUI, ranging from 0.5 to 5), plant part used (PUI, ranging from 0.75 to 2), multifunctional food use (MFFI, ranging from 0.5 to 2), taste score appreciation (TSAI from 4 to 10), and food/medicinal role (FMRI, ranging from 1 to 5) (see Pieroni 2001).

The significance of the differences between samples was calculated using the Mann–Whitney *U* test. Statistics were calculated using the open access statistical package PAST (Hammer *et al.*^2001^; PAST 2012), apart from Salience Index and CFSI, which were calculated using the formula entered into Microsoft Excel®.

## Results

### Use of Wild Edible Plants by the Polish Community

The uses of wild edible plants reported by informants are mainly contemporary, i.e., referring to the last 5 years (73 % of use-reports). We recorded the use of 41 botanical species and two mushroom taxa among Polish migrants and their descendants (Table 1). These species belong to 26 botanical families, of which the largest are Myrtaceae (8 species), Arecaceae (3), Asteraceae (3) and Rosaceae (3).

**Table 1:** Wild food plants and fungi used by the Polish community in Misiones, Argentina

Family	Scientific name, local name	Part used	Mode of consumption	Habitat	Habit	Origin	Citation index (CI)	Salience index (SI)	Cultural food signifi-cance index (CFSI)
Annonaceae	*Rollinia salicifolia* Schltdl., araticú, areticú	Fruit	Raw, as snack fruit	fe, for, fd, ps, gar	Tree	Native	44	0.670	6.23
Araceae	*Philodendron bipinnatifidum* Schott, güembé, banana del monte	Fruit	Raw, as snack fruit	fe, for, rud, fd, ps	Epiphyte	Native	20	0.208	2.3
Araucariaceae	*Araucaria angustifolia* (Bert.) O.K., pino paraná, pino araucaria	Seeds	Roasted	fe, for, fd	Tree	Native	3	0.038	0.46
Arecaceae	*Acrocomia aculeata* (Jacq.) Lodd., coco	Fruit	Raw, as snack fruit	fe, for, ps, fd, gar	Palm	Native	7	0.078	0.88
Arecaceae	*Euterpe edulis* Mart., palmito	Palm heart	Cooked or fried, in salad, stuffing for pastry	for	Palm	Native	17	0.182	6.8
Arecaceae	*Syagrus romanzoffiana* (Cham.) Glassman, pindó	Fruit, young shoots	Raw, as snack fruit; in salad	fe, for, rud, fd, ps	Palm	Native	49	0.469	7.8
Asteraceae	*Hypochaeris chillensis* (Kunth) Hieron., achicoria silvestre, achicoria salvaje, dzika achicoria	Leaves	Raw, in salad	rud, fd, gar	Herb	Native	39	0.264	68.23
Asteraceae	*Lactuca virosa* L., lechuga brasilera	Leaves	Raw, in salad	fd	Herb	Alien	3	0.008	1.97
Asteraceae	*Sonchus oleraceus* L., diente de león, amargón, borraja, mlicz	Leaves	Raw, in salad	rud, fd, ps	Herb	Alien	3	0.041	2.13
Brassicaceae	*Lepidium didymum* L., mintruiz, mistruiz	Aerial parts	Raw, in salad	rud, fd, gar	Herb	Native	7	0.051	6.12
Brassicaceae	*Nasturtium officinale* R. Br., berro de agua	Aerial parts	Raw, in salad	wat, ps	Herb	Alien	9	0.038	5.97
Bromeliaceae	*Ananas comosus* (L.) Merr., ananá del monte	Fruit	Raw, as snack and dessert fruit	fe, for, fd	Succulent	Alien	3	0.053	0.22
Cactaceae	*Opuntia arechavaletae* Speg., tuna	Fruit	Raw, as snack fruit	fe, rud, ps	Succulent	Native	4	0.013	0.16
Caricaceae	*Carica papaya* L., mamón	Fruit	Raw, as dessert fruit; jam	fe, sec, fd, gar	Tree	Native	6	0.088	25.13
Caricaceae	*Jacaratia spinosa* (Aubl.) A. DC., yacarati’a, yaracati’a, mamón del monte	Fruit	Raw, roasted, as snack fruit	for	Tree	Native	19	0.214	0.29
Celtidaceae	*Celtis iguanaea* (Jacq.) Sarg., tala	Fruit	Raw, as snack fruit	fe, for, rud, ps	Tree	Native	14	0.117	0.94
Clusiaceae	*Rheedia brasiliensis* (Mart.) Planch. & Triana, pacurí	Fruit	Raw, as snack fruit	for	Tree	Native	7	0.083	0.08
Cucurbitaceae	*Cyclanthera tenuisepala* Cogn., pepinillo, pepinito (del monte)	Fruit	Raw, lacto-fermented, as snack fruit, in salad	fe, rud, fd	Creeper	Native	5	0.036	0.57
Fabaceae	*Inga affinis* DC., inga	Fruit	Raw, as snack fruit	fe, for, gar	Tree	Native	5	0.040	0.36
Lamiaceae	*Vitex megapotamica* (Spreng.) Moldenke, tarumá	Fruit	Raw, as snack fruit	for, wat	Tree	Native	4	0.040	0.1
Malpighiaceae	*Dicella nucifera* Chodat, maní del mono, maní del monte	Fruit	Raw and roasted, as snack fruit	fe, for, rud	Liana	Endemic	6	0.040	1.08
Moraceae	*Morus alba* L., mora	Fruit	Raw, as snack and dessert fruit; jam; liqueur	fe, for, rud, ps, gar	Tree	Alien	17	0.148	1.98
Myrtaceae	*Campomanesia guazumifolia* (Cambess.) O. Berg, sietecapote, sietecapotes	Fruit	Raw, as snack fruit	fe, for, ps, fd, gar	Tree	Native	20	0.198	1.26
Myrtaceae	*Campomanesia xanthocarpa* Mart. ex O. Berg, guavirá	Fruit	Raw, as snack fruit; jam	fe, for, ps, fd, gar	Tree	Native	40	0.508	4.91
Myrtaceae	*Eugenia involucrata* DC., cerella, cereza, wiśnia, dzika czereśnia	Fruit	Raw, as snack fruit; liqueur; jam	fe, for, wat, fd, ps, gar	Tree	Native	46	0.681	3.27
Myrtaceae	*Eugenia pyriformis* Cambess., uva jay	Fruit	Raw, as snack fruit, cooked as compote	fe, sec, wat, ps	Tree	Native	31	0.332	2.38
Myrtaceae	*Eugenia uniflora* L., pitanga	Fruit	Raw, as dessert and snack fruit; liqueur	fe, for, wat, fd, ps, gar	Shrub or tree	Native	47	0.698	11.29
Myrtaceae	*Plinia rivularis* (Cambess.) Rotman, guaporait’í, vaporait’í, vaporit’í	Fruit	Raw, as snack fruit	fe, for, wat, gar	Tree	Native	21	0.263	0.45
Myrtaceae	*Plinia peruviana* (Poir.) Govaerts, yaboticaba	Fruit	Raw, as snack and dessert fruit; compote; liqueur	for, gar	Tree	Native	22	0.266	3.6
Myrtaceae	*Psidium* spp., guayava	Fruits	Raw, as snack and dessert fruit; jam	fe, for, wat, rud, fd, ps	Shrub or tree	Native	22	0.225	3.18
Oxalidaceae	*Oxalis debilis* Kunth, batatita, tipo trébol, szczaw	Leaves, bulb	Raw, as snack; soup	fe, for, wat, rud	Herb	Native	7	0.026	0.59
Passifloraceae	*Passiflora alata* Curtis, maracuyá	Fruit	Raw, as snack fruit; juice	fe, secondary forest, rud, gar	Liana	Native	3	0.031	0.97
Plantaginaceae	*Plantago australis* Lam., llantén	Leaves	Raw, in salad	rud, gar	Herb	Native	2	0.021	9.18
Polygonaceae	*Rumex paraguayensis* D. Parodi, szczaw, lengua de vaca	Leaves	Raw, in salad; soup	wat, rud, fd, gar	Herb	Endemic	8	0.064	1.75
Rhamnaceae	*Hovenia dulcis* Thunb., hovenia	Fruit	Raw, as snack fruit; refreshing drink; liqueur	fe, sec, rud, fd, ps, gar	Tree	Alien	24	0.163	2.44
Rosaceae	*Eriobotrya japonica* (Thunb.) Lindl., níspero	Fruit	Raw, as snack fruit; cooked as compote; jam	fe, sec, fd, gar	Tree	Naturalized	4	0.035	4.1
Rosaceae	*Rubus rosifolius* Sm., frambuesa silvestre, frutilla del monte, maliny, malinki, drapaki	Fruit	Raw, as snack fruit, as dessert fruit with cream and sugar; jam	fe, rud, wat, ps, fd	Shrub	Alien	19	0.141	2.3
Rosaceae	*Rubus sellowii* Cham. & Schltdl., mora, mora del monte, jeżynki, drapaczyna	Fruit	Raw, as snack fruit	fe, for, rud, ps, fd	Shrub	Endemic	7	0.037	0.62
Rutaceae	*Citrus aurantium* L., apepú	Fruit	Raw, as condiment, juice, refreshing drink	for, ps	Tree	Alien	3	0.005	1.31
Sapindaceae	*Allophylus edulis* (A. St.-Hil., A. Juss. & Cambess.) Hieron. ex Niederl., cocú	Fruit	Raw, as snack fruit; liqueur	fe, for, rud, ps, fd, gar	Shrub or tree	Native	33	0.336	6.41
Solanaceae	*Solanum sisymbriifolium* Lam., espina colorada, jua	Fruit	Raw, as snack fruit	fe, rud, ps, fd	Subshrub	Native	3	0.032	0.2
Agaricaceae (Mushrooms)	*Agaricus* sp., pieczarki	Fruiting body	Fried	ps	Fungus	Alien	2	0.050	0.27
Suillaceae (Mushrooms)	*Suillus* sp., hongos, grzyby	Fruiting body	Fried	for	Fungus	Native	2	0.010	0.24

Habitat: *fd* fields, *fe* forest edges, *for* primary and secondary forests, *gar* home gardens, *ps* pastures, *rud* ruderal areas, *sec* secondary forests, *wat* waters

Interviewees: in total 50 persons: Wanda colony (35), Lanusse colony (15)

Thirty-one species (76 %) are used for their edible fruits. They are usually consumed in the form of raw snacks, and to a lesser extent as dessert fruits, juices, compotes, jams and liqueurs. Seven botanical taxa (17 %) are used for their leaves and young shoots. They are consumed mainly in salads, soups, lacto-fermented dishes, and also eaten raw as snacks. One genus is used for its edible tubers (*Oxalis debilis* Kunth), one species for its palm hearts (*Euterpe edulis* Mart) and another for its seeds (*Araucaria angustifolia* (Bert.) O.K.).

**Fig. 2:**
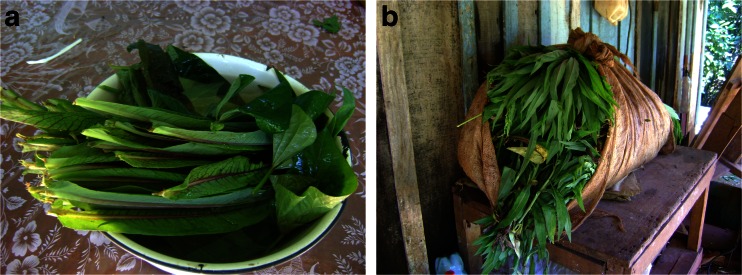
**a**) *Lactuca virosa* and *Ipomoea batatas* leaves used in salad by Polish descendants in Lanusses, Northern Misiones, Argentina; **b**) A bundle of *Lactuca virosa* leaves (Lanusse, 15.03.2011, Monika Kujawska)

None of the recorded species have ever been used as famine food, as Polish settlers in Misiones have never suffered from a severe food shortage. Nevertheless, a few species were used as emergency food, especially in the past when there was less access to manufactured products and unseasonal vegetables. Wild green vegetables such as *Hypochaeris chillensis* (Kunth.) Hieron., *Lactuca virosa* L. and *Lepidium didymum* L., which grow in the wintertime, have been used as emergency greens in salads. Young leaves of the cultivated sweet potato *Ipomoea batatas* (L.) Lam. were reported as used for the same purpose because Polish settlers wanted ‘something green’ to accompany meat and cassava (Fig. 2). Another example of emergency food is the use of *Cyclanthera tenuisepala* Cogn. fruits as lacto-fermented cucumbers, as reported by three families. One informant described the way her family learned about wild greens:

The Italians used to come; there [in Brazil] was one colony comprised exclusively of Italians (…) They would come to our house and look for *achicoria silvestre* [*Hypochaeris chillensis*], because it grows only in the winter (…) and this is how we learned from them. But my grandmother and my mother-in-law who were from Poland always searched for it and prepared salad (…) Because when they arrived they did not have gardens (…) they looked for something to make salad. *Szczaw* [*Rumex* spp.] they ate too. (…) My mother-in-law told me that they would cut it and make soup with it, adding flour and cow fat. And *mistruz* [*Lepidium didymum*] (…) these Italians used to come and cut it. One day I said to my mother: “Let’s try it!” (…) I washed it well and cut it finely, and it is delicious, but it has this strong taste. (Woman, 64, Wanda).

Among reported species, trees predominate (18 species, 44 %), followed by herbs (9 species, 22 %), then shrubs and sub-shrubs (7 species, 17 %), palms (3), lianas (2), creepers (1) and epiphytes (1). Most wild edible plants used by Polish migrants and their descendants are native to the area (31 species, 76 %), and three species are endemic (*Dicella nucifera* Chodat, *Rumex paraguayensis* D.Parodi and *Rubus sellowii* Cham.&Schltdl.). Adventitious and naturalized plants are also used (10 species, 24 %). Alien species are represented mainly by herbaceous ruderal plants (*Lactuca virosa* L., *Nasturtium officinale* R. Br, *Sonchus oleraceus* L.) and fruit trees (*Morus alba* L., *Hovenia dulcis* Thunb., *Eriobotrya japonica* (Thunb.) Lindl., *Citrus aurantium* L).

The phytonyms used by the Polish community reflect the multicultural character of the border region where they live. Most of the appellatives are of Spanish origin, but some come from the Guarani (*pindó*, *yacarati’a*, *tarumá*, *pacurí*, *guaporait’í*, *apepú*, *güembé*, *uva jay*, *inga*, *maní* (del monte), *cocú*, etc.). For other species there are pairs of Spanish and Polish appellatives: *lengua de vaca* and *szczaw*, *tipo trebol* and *szczaw*, *frambuesa del monte* and *maliny* / *malinki*, *mora* and *jeżynki* / *drapaczyna*. These Polish names have survived the migration process and were given to plants that resembled species known from Poland (belonging to the same botanical genera).

### Cultural Significance of Wild Edible Plants

According to the number of reports (Citation Index) the most culturally significant species are wild native fruits (Table 1): *Syagrus romanzoffiana* (Cham.) Glassman (49 citations), *Eugenia uniflora* L. (47), *Eugenia involucrata* DC. (46), *Campomanesia xanthocarpa* Mart. (40), *Allophylus edulis* (A. St.-Hil., A. Juss. & Cambess.) Hieron. ex Niederl (33). The only wild green vegetable that achieved a large number of citations was a native ruderal species, *Hypochaeris chillensis* (Kunth) Hieron. (39 citations).

The Salience Index gave similar results to the Citation Index. Fruits stand out as culturally salient wild food for Polish migrants and their descendants. The most culturally important species are *Eugenia uniflora* L. (0.698), *Eugenia involucrata* DC. (0.681), *Rollinia salicifolia* Schltdl. (0.670), *Campomanesia xanthocarpa* Mart. ex O. Berg (0.508), *Syagrus romanzoffiana* (Cham.) Glassman (0.469), etc. The only green vegetable in the first 10 most salient species was *Hypochaeris chillensis* (Kunth) Hieron. (0.264), in eighth position.

On the other hand, the Cultural Food Significance Index yielded different results. The most culturally significant plant according to this index is a wild green, *Hypochaeris chillensis* (Kunth) Hieron. (68.23), followed by *Carica papaya* L. (25.13) (with only 6 reports), *Eugenia uniflora* L. (11.29), *Plantago australis* Lam. (9.18) (with only 2 reports), and *Syagrus romanzoffiana* (Cham.) Glassman (7.8). Generally, wild greens achieved higher values in this index than when using the Citation or Salience indices to measure the cultural importance of wild edible plants.

### Sociodemographic Factors

**Fig. 3:**
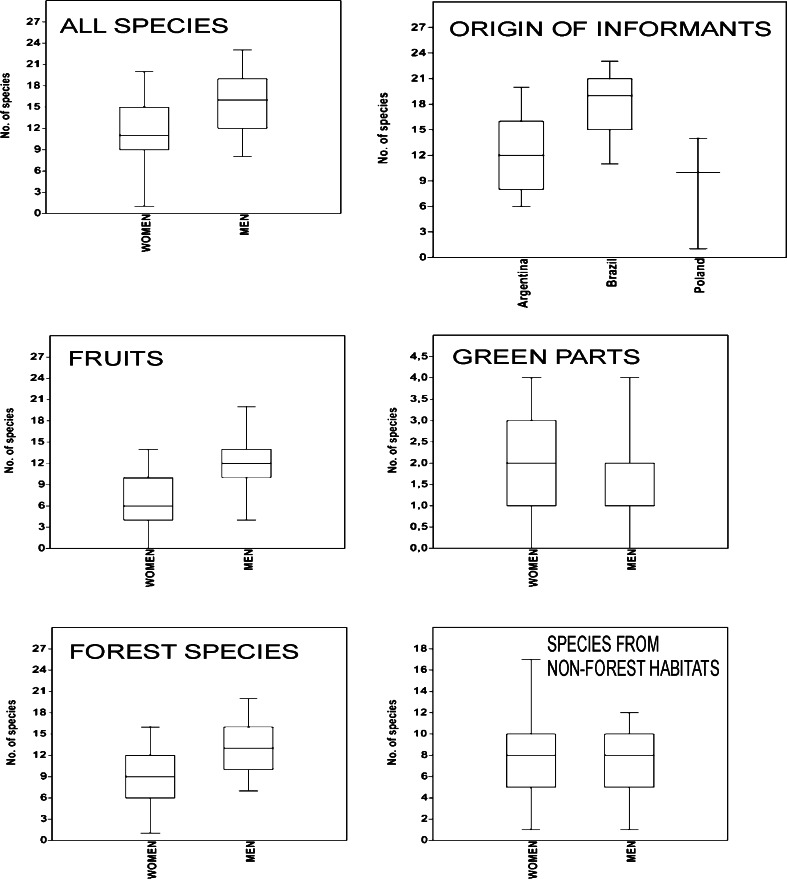
Differences in use of wild edible plants based on gender, habitat preference and origin (Argentina—offspring of Polish migrants from the first and second generation, Brazil—descendants of Poles who migrated to Argentina via Brazil; Poland—Polish migrants who migrated to Argentina as children). The *box plot* shows minima, maxima, first and third quartile and median

The study participants reported on average 13.3 ± 4.7 SD (median – 13) wild species, among them 11.1 fruits (median – 11.5) and 1.6 green vegetables (median – 1.5). However, the knowledge and use of wild food resources is not equally distributed in the Polish community. Women reported on average 11.5 ± 4.3 taxa (median – 11) and men reported on average 15.3 ± 4.4 botanical species (median – 16) (Fig. 3). This difference between sexes is statistically significant (*p* = 0.0068; Mann–Whitney *U* test). On the other hand, there was no clear age effect (neither using linear regression nor fitting a unimodal curve gave statistically significant results).

There are significant differences in knowledge depending on the migrants’ origin. The descendants of Poles who first migrated to Brazil at the end of the nineteenth century (and their children and grandchildren who moved to Misiones between 1930 and 1950s) displayed the most diversified knowledge about wild edible resources. These people represent the second and the third generation born in South America and reported on average 18.0 ± 3.8 botanical species. The second most knowledgeable group comprises descendants of Polish migrants mostly from the first and occasionally from the second generation born in Argentina, mentioning on average 12.0 ± 3.9 wild edible species. The informants that had the poorest knowledge about wild edible resources were Polish pioneers who migrated with their parents from Eastern Poland to the northern Misiones as children. They elicited on average 9.0 ± 4.9 wild food plants (Fig. 3). The differences between any of the pairs of these three migration categories were statistically significant (*p* < 0.05; Mann–Whitney *U* test).

Wild plant resources are gathered from different environments, such as primary and secondary forest, forest edge, surroundings of streams and rivers, pasture, farmland, ruderal areas, home gardens and household surroundings. For the purpose of analysis we lumped them into two habitat types: ruderal areas and forests. Women mentioned on average 8.0 ± 3.7 species from ruderal areas, whereas men reported 7.3 ± 2.9, thus the difference in the exploitation of ruderal zones was not statistically significant between genders (Fig. 3). On the other hand, men knew and used more species from primary and secondary forests (*p* = 0.0004; Mann–Whitney *U* test). They mentioned on average 11.9 ± 4.5 species, while women elicited only 6.9 ± 3.9 forest species (Fig. 3). Moreover, the use of forest edible plants refers to contemporary uses in case of men, while women remembered the forest species mostly from the past, when as children they would explore the forest in the company of other children during siesta hours and holidays.

### Wild Edible Plants – Not Considered as Medicines

Generally the Polish community of Misiones does not perceive wild edible fruits as healthy food resources. Fruits are eaten mainly as snacks because they have an interesting taste that differs from cultivated plants. However, some fruits are sporadically consumed for their healing qualities: *Carica papaya* L., which, according to informants, has laxative properties, *Passiflora alata* Curtis consumed for its sedative properties and *Citrus aurantium* L., whose juice is applied in the treatment of respiratory system illnesses. On the other hand, wild greens are regarded as healthy food, especially *Hypochaeris chillensis* (Kunth) Hieron., *Sonchus oleraceus* L., *Lepidium didymum* L., *Nasturtium officinale* R. Br. and *Plantago australis* Lam. Nevertheless, for the majority of the recorded taxa we could not establish a link between their nutritional and medicinal properties.

## Discussion

### Wild Food Plants in the Migratory Process

Only one species from the list provided by Polish migrants and their descendants was known from their country of origin, namely *Sonchus oleraceus* L. However, very few participants could remember its Polish dialect name—*mlicz*. We have little information from Poland about the use of *Sonchus oleraceus* L., which is probably due to the fact that Polish farmers were not in the habit of employing wild herbs in salads, especially bitter tasting ones (Łuczaj and Szymański 2007; Łuczaj 2008, 2010). Polish pioneers most likely learned to employ greens in salads from other migrants, for example, Italians and Germans or from mestizo people who had contact with Spanish colonists. It may have been also due to the influence of Mbya Guarani people from Misiones who use different species of *Hypochaeris* in salads or in potherbs (Martínez-Crovetto 1968). Nevertheless, Polish migrants found plants in Misiones from the same botanical genera as a few wild food species known from Poland, e.g., *Oxalis debilis* Kunth, *Rumex paraguayensis* D. Parodi, *Plantago australis* Lam., *Rubus rosifolius* Sm. and *Rubus sellowii* Cham. & Schltdl., and most of these plants received Polish names. In Poland, *Oxalis acetosella* L.*, Rumex acetosa* L. and numerous *Rubus* species have been widely used as food plants. However, the related species in Argentina are not popular among the descendants of Polish settlers. The predilection for fruits displayed by Polish pioneers was probably enhanced in Misiones by the fact that wild edible fruits are abundant, diverse and easily accessible resources there. Since wild edible fruits are also important for local people (Martínez-Crovetto 1968), their use was strengthened during extra-ethnic contacts. This must have had a positive impact on the consistency of consumption preference for wild fruits.

At the same time the cultural preference for a sour taste (and the importance of sorrel soup) declined during the migratory process. However, we are not aware of any explanation for this process. Poles in Misiones were receptive to new food and patterns of consumption influenced by local people (both mestizo and indigenous groups). They quickly switched from potatoes to cassava, from pork to beef and from tea to yerba mate, because of their availability and lower prices (Kujawska 2010). Sorrel is not consumed in the form of soup by mestizo or indigenous peoples of Misiones, so even as an important traditional dish, it did not survive the acculturation process. Moreover, *Rumex paraguayensis* D. Parodi, although morphologically similar to the *Rumex* species known from Poland, was not adopted by Polish migrants due to its rather dull taste, less sour than that of sorrel (*R*umex *acetosella* L.). Therefore, we may assume that it is not the morphological similarity of plants found in the new environment that favors their use by an émigré community, but rather a taste that is similar to known and preferred foods.

Remarkably, the study population utilizes only two taxa of fungi. A large number of edible mushrooms is gathered in all regions of Poland (Łuczaj and Nieroda 2011), usually at least fifteen taxa. The large reduction in gathered taxa may arise from the reduced availability of edible mushrooms and the fear of poisoning in the new environment.

### Study of Wild Edible Plants in Argentina in General, and in Misiones in Particular

There is only one published study on wild edible plants in Misiones, conducted among Mbya Guarani Indians (Martínez-Crovetto 1968). It points out the importance of wild fruits for the indigenous people of Misiones. Most fruits used by Polish migrants and their descendants are found on the list provided by Martínez-Crovetto (1968). However, the Mbya use more wild fruit species than the Polish community does, as many as 60 wild fruits (Martínez-Crovetto 1968: 10–11), compared to 31 taxa of wild fruits identified in our study. Other studies from Argentina, especially from Chaco (Arenas and Scarpa 2007) and the central part of the country (Cordoba province) (Arias Toledo *et al.*^2007^) also indicate the dominance of wild fruits over other wild food plant categories among the indigenous and mestizo populations. Green vegetables prevail over other plant parts and food types only in the Patagonian region (Ladio and Lozada 2003, 2004). The number of species recorded in other parts of Argentina is similar or even lower than among the Polish community from Misiones. Arenas and Scarpa (2007) recorded the food use of 57 botanical species among the Chorote from Chaco, Arias Toledo and collaborators reported on the use of 25 wild species among the mestizo population from Cordoba province, and Ladio and Lozada (2004) found 42 wild plants among the Mapuche indigenous people from the Northwestern Patagonia. These figures show that different ethnic groups in Argentina, and in the South Cone in general, use natural resources in their diet in a moderate way. Polish migrants and their descendants share with other ethnic Argentinean groups, especially from Patagonia and central Argentina, the use of exotic wild greens such as *Sonchus oleraceus* L., *Nasturtium officinale* R. Br. (Ladio and Lozada 2003, 2004) and plants of the same botanical genera as European species such as *Oxalis*, *Hypochaeris*, and *Rumex*, which represent both native and alien species (Lozada *et al.*^2006^; Arias Toledo *et al.*^2007^).

Based on our data, we decided to make a subtle distinction between emergency food and famine food. Our research participants claimed that they had never suffered from severe hunger in Argentina, compared to their ancestors in Poland. Therefore, we assumed neither the wild greens nor the wild fruits mentioned during interviews could be considered as famine food for this community. However, a few species, especially wild green vegetables, have been employed in times of temporary food shortage (mainly winter time), as a kind of less-favored food, eaten on an infrequent basis.

Nevertheless, we acknowledge that emergency food may become famine food under certain circumstances. This usually happens when marginally edible wild plants in a given local group are eaten on a regular basis during a prolonged food shortage, due to a recurrence of bad crops, or social unrest such as war. Therefore, sometimes the distinction between these two types of food is more a matter of degree than a total behavioral change (see also Huss-Ashmore and Johnston 1994; Johns 1994).

A lack of famine food plants is not the only characteristic of the Polish community in Misiones. Another is the complete lack of wild species used for recreational teas (see Sõukand *et al.*^2013^). In the same fashion, we were not able to record any food additives used as condiments, spices and seasonings, apart from the sporadic use of *Citrus aurantium* L. fruit juice as a substitute for lemon in salads. The lack of wild plants employed as teas can be explained by the fact that the most important drink in the region is an infusion of yerba mate and its cold version—*tereré*. Therefore, many wild plant species are added to improve the taste of mate and tereré, as well as being used as a prophylactic. These plants, however, were not elicited during field research dedicated to wild edible plants, as they were clustered with medicinal plants (Kujawska and Hilgert 2014; Kujawska and Pieroni 2015). A strong reduction in the use of recreational herbal teas was also observed in Poland and Russia throughout the nineteenth and twentieth centuries due to the popularity of black tea (Sõukand *et al.*^2013^). Thus one might conclude that the people who arrived in South America were already out of the habit of drinking herbal teas.

### Cultural Significance Indices

Many researchers have underlined the advantages of quantitative ethnobotanical indices as valuable tools for comparing knowledge and uses of natural resources between different environments and different ethnic groups (Philips and Gentry 1996; Ladio and Lozada 2003; Silva and Andrade 2006; Sousa Araújo *et al.*^2012^; Mathur and Sundaramoorthy 2013).

In this article, we applied three different indices to measure the cultural significance of wild edible plants among the Polish community in Misiones. The Citation Index and the Salience Index yielded similar results, highlighting the importance of wild fruits for this community and this particular region. However, the Cultural Food Significance Index (CFSI) (Pieroni 2001) produced very different results, exposing the importance of wild greens. These differences stem from the fact that the CFSI favors wild green vegetables, by giving more points to green parts of wild plants than to fruits. As a consequence, in our opinion, the CFSI overestimates the importance of wild green vegetables in local diets. In our study, among the ten most important wild food plants (with the highest CFSI values) there are five wild green vegetables, although the majority of these species have few citations and low salience index scores. For example, *Plantago australis* Lam., with only two citations, has a higher CFSI value than *Syagrus romnazoffiana* (Cham.) Glassman, which had 49 citations (nearly all participants). In the study performed by Pieroni (2001), wild green vegetables also achieved the highest scores, with wild greens being highly appreciated resources in the Mediterranean region, as confirmed by other research (Leonti *et al*. 2006). In our study region, wild fruits stand out as typical and highly appreciated wild food, but the CFSI index underplays their values by giving fruit (plant part) and fruit snacks less points than other food types and plant parts used. Therefore, it can be concluded that this index is very region-specific and culture-sensitive, and therefore appropriate for the analysis of wild edible plants in the Mediterranean but less suitable for the study of wild foods in the subtropics and tropics.

Moreover, higher CFSI values were achieved by species that had some medicinal properties. For example *Plantago australis* Lam*.* (two Citations) scored 9.18 CFSI. *Carica papaya* L., with only six citations scored the second rank in CFSI importance. This stems from the fact that the CFSI gives additional points to edible species with medicinal properties. However, we argue that this index should not pay so much attention to the medicinal aspects of wild foods.

Pieroni’s CFSI, after some modifications, has been used to evaluate the cultural significance of mushrooms in Mexico (Garibay-Orijel, *et al.*^2007^; Alonso-Aguilar *et al.*^2014^). The modifications introduced (not distinguishing the parts of the mushrooms) are probably responsible for the fact that the discrepancy between its scores and the Citation Index are not large, in contrast to our studies.

Among the studied species, only *Eugenia uniflora* L. achieved a high number of citations (47), a high salience value (0.698) and relatively high CFSI rank (11.29). In our opinion, this species can be considered as the most important and appreciated wild edible plant for the Polish community in Misiones. It is a very ubiquitous plant, often grown in home gardens as a managed or protected species. Therefore, its popularity stems from plant availability, as well as its taste, which is highly appreciated. None of the wild edible plants can be considered as cultural markers of identity for the Polish community in Misiones, based on ethnic grounds, but *Eugenia uniflora* L. can be considered the most salient species.

### Sociodemographic Differences in Use of Wild Edible Plants

The sociodemographic differences in knowledge distribution found in this study have been confirmed by other research from Argentina, Poland and elsewhere. In the Argentinean Chaco, amongst the Chorote people, there are women who dedicate themselves to searching and preparing wild edible plants (Arenas and Scarpa 2007). The same pattern has been observed in Cordoba province among the mestizo residents (Arias Toledo *et al.*^2007^). Similarly, in the Polish countryside there were mainly women and children who dedicated themselves to plant and mushroom gathering (Łuczaj 2008; Łuczaj and Nieroda 2011). This activity appears to have undergone changes in Misiones among the Polish group, as nowadays men have a wider knowledge of wild food plants, use a greater number of taxa, and know more species from the forest. This is due to the fact that men move within a wider spectrum of herbal landscape (sensu *lato* Sõukand and Kalle 2010). Their memory and identification skills are constantly trained during work in the forest, the forested parts of their land parcels, and in the pastures. At the same time, women confine their movements within the herbal landscape to homegardens, farmland, and ruderal areas. Due to this division, men definitely know wild fruits better than women, who have a poorer knowledge of fruits but a slightly better knowledge of wild greens, which they occasionally employ as substitutes for lettuce (*Lactuca sativa* L.) and chicory (*Cichorium intybus* L.). A higher level of knowledge of wild foods among men was also observed in central China (Kang *et al.*^2013^), also due to the larger scope of male mobility.

The use of the greatest number of wild edible species can be found among the descendants of Polish migrants who came to Argentina via Brazil. Migrants came from the Polish countryside, which in the nineteenth century was still dominated by the feudal system, and once in Brazil they settled in rural areas. The push and pull factors of this migration were purely economic. According to Bonasewicz (2000), Poles from the first wave of migration (the end of the nineteenth and the beginning of the twentieth centuries) showed the strongest resistance towards acculturation and integration within Brazilian and Argentinean society. This differs in comparison with the migrants from the second wave (1920s–1930s) and from the third wave after World War II. Polish pioneers from the first wave of migration lived in thoroughly homogenous settlements, intermarried, retained a family pattern of farm work and conserved their native language (Bonasewicz 2000). They had to develop knowledge about the local flora and fauna quickly, as they lived in isolated colonies, far away from city centers, shops and health care providers. These cultural circumstances explain why descendants of Poles who first migrated to Brazil and later moved on to northern Misiones retained the Polish language and some native customs but also gained a thorough familiarity with the local flora and fauna. They have been resident the longest in South America, display confidence in their knowledge of local flora, and move with ease within the local herbal landscape.

## Conclusions

Wild fruits are the most appreciated resources and the most salient species used by the Polish community in Misiones. The cultural importance of wild fruits for Polish migrants was strengthened through their diversity and easy availability throughout Misiones. Their relevance was also reinforced in extra-ethnic relations with other groups of Misiones: the mestizos and Mbya Guarani indigenous people.

Green vegetables, on the other hand, are of little importance among the study group, although they do not have connotations of poverty food, as was the case in Poland and in other parts of Argentina. Their reduced use stems rather from the fact that wild greens have never been appreciated by Polish migrants and their use has not been reinforced through extra-ethnic contacts.

An interesting feature of the Polish migrant community and their descendants in Misiones is that they confine the use of wild resources to wild fruits and a few species of wild green vegetables. They never prepare recreational teas or make condiments out of wild plants. Nor have any of the species ever been employed as famine food, as this community has no memories of severe periods of food shortage.

Within the study community, there are marked differences in the use of wild food plants based on the gender and the origins of Polish migrants and their descendants. Within the migratory process, men have acquired a larger cultural competence in obtaining wild food plants from a greater number of habitat types than women, who confine their movement within the herbal landscape to home gardens and ruderal areas. One of the main conclusions of our research is that the longer Polish descendants stay in South America, the richer and more diversified their knowledge about wild edible plants becomes.

## Footnotes

1 Enculturation is the process through which an individual acquires cultural knowledge and assimilates cultural values.

2 But see Pieroni (2003) and Tizio *et al.* (2012) for research in the Mediterranean region among Albanian and South-Slavic migrants, and Pieroni *et al.* (2012) among Italians in Romania, and Volpato *et al.* (2009) for studies of Haitian migrants in Cuba.
